# The effects of aerobic, resistance, and meditative movement exercise on sleep in individuals with depression: protocol for a systematic review and network meta-analysis

**DOI:** 10.1186/s13643-019-1018-4

**Published:** 2019-04-26

**Authors:** Gavin Brupbacher, Heike Gerger, Monika Wechsler, Thea Zander-Schellenberg, Doris Straus, Hildburg Porschke, Markus Gerber, Roland von Känel, Arno Schmidt-Trucksäss

**Affiliations:** 1 Division of Sports and Exercise Medicine, Department of Sport, Exercise and Health, University of Basel, Birsstrasse 320 B, 4052 Basel, Switzerland; 2 Oberwaid AG, Rorschacher Strasse 311, 9016 St. Gallen, Switzerland; 3 Division of Clinical Psychology and Psychotherapy, Faculty of Psychology, University of Basel, Missionsstrasse 62a, 4055 Basel, Switzerland; 4 University Medical Library Basel, Spiegelgasse 5, 4051 Basel, Switzerland; 5 Division of Clinical Psychology and Epidemiology, Faculty of Psychology, University of Basel, Missionsstrasse 62a, 4055 Basel, Switzerland; 6 Division of Sport and Psychosocial Health, Department of Sport, Exercise and Health, University of Basel, Birsstrasse 320 B, 4052 Basel, Switzerland; 7 Department of Consultation-Liaison Psychiatry and Psychosomatic Medicine, University Hospital Zürich, University of Zurich, Culmannstrasse 8, 8091 Zurich, Switzerland

**Keywords:** Exercise, Aerobic, Resistance, Meditative movement, Depression, Sleep, Randomized, Trial, Systematic review, Network meta-analysis, Protocol

## Abstract

**Background:**

The main objective of this review is to assess the effects of aerobic, resistance, and meditative movement exercise on sleep quality in patients with unipolar depression. A secondary goal is to ascertain the effects on sleep duration, sleepiness, daytime functioning, use of hypnotics, and adverse events.

**Methods:**

A systematic computerized search will be performed in the following online databases: PubMed, EMBASE (on Ovid), Cochrane Library, PsycINFO (on Ovid), SPORTDiscus (on EBSCOhost), CINHAL (on EBSCOhost), Clinicaltrials.gov, WHO International Clinical Trials Registry, OpenGrey, and ProQuest Dissertations and Theses. Bibliographies of all included studies as well as any other relevant reviews identified via the search will be screened. Randomized trials using aerobic, resistance, or meditative movement exercise interventions which target sleep as a primary or secondary outcome will be included. The primary outcome will be differences in sleep quality at post-intervention. Secondary outcomes will be adverse events, differences in sleep duration, daytime sleepiness and functioning, and the use of hypnotics at post-intervention. Two authors will independently screen the identified records. Disagreement will be resolved by consensus or if no consensus can be reached by adjudication of a designated third reviewer. Data extraction will be done independently by two authors using a standardized and piloted data extraction sheet. Bias in individual studies will be assessed using the revised Cochrane risk of bias tool. The certainty of evidence across all outcomes will be evaluated using the CINeMA (Confidence in Network Meta-Analysis) framework. A frequentist network meta-analysis will be conducted. The systematic review and network meta-analysis will be presented according to the PRISMA for Network Meta-Analyses (PRISMA-NMA) guideline.

**Discussion:**

This systematic review and network meta-analysis will provide a synthesis of the currently available evidence concerning the effects of aerobic, resistance, and meditative movement exercises on sleep in patients with unipolar depression. Thereby, we hope to accelerate the consolidation of evidence and inform decision-makers on potential benefits and harms.

**Systematic review registration:**

The protocol has been registered at the International Prospective Register of Systematic Reviews (PROSPERO; registration number: CRD42019115705).

**Electronic supplementary material:**

The online version of this article (10.1186/s13643-019-1018-4) contains supplementary material, which is available to authorized users.

## Background

### Description of the condition

Worldwide lifetime prevalence of unipolar depression is estimated to be 10% [1]. Unipolar depression is the leading cause of burden of disease in middle- and high-income countries and is projected to become the leading cause worldwide by 2030 [2]. The economic burden of unipolar major depression alone was estimated to be $210 billion in the USA in 2010 [3]. In addition to the direct and often debilitating symptoms, unipolar depression also substantially increases the risk of all-cause mortality [4] and morbidity [5].

One of the diagnostic criteria for depression according to DSM-V [6] and ICD-10 [7] is disturbed sleep, i.e., insomnia or hypersomnia. This is reflected by the high prevalence (up to 90%) of co-occurring insomnia in individuals with depression [8]. Insomnia has a negative impact on health-related quality of life and daytime functioning [9, 10]. Moreover, sleep disturbances have been recognized as a mechanistic (i.e., causal or bidirectional) process in depression. Sleep problems are an independent risk factor for depression [11]. Depression and insomnia are linked in a bidirectional manner [12]. Comorbid sleep disorders have a negative influence on the disease trajectory in depressive disorders [13]. Some of the most prevalent residual symptoms after treatment response or remission in this clinical population are insomnia symptoms [14, 15]. This is pertinent because persistent sleep disorders increase the likelihood of relapse [13, 16, 17]. Sleep disorders are an independent risk factor for suicide [18, 19].

According to the British national (NICE) treatment guidelines, individuals with depression are to be treated with psychotherapy and pharmacotherapy [20]. However, antidepressants are of limited efficacy [21] and can cause considerable adverse effects. In a primary care setting the number needed to treat (NNT) for improvement of depressive symptoms by selective serotonin reuptake inhibitors is approximately seven whereas the number needed to harm (NNH) (withdrawal due to side effects) is estimated to be between 20 and 29 [22]. A combination of antidepressants and benzodiazepine receptor agonists (Z-drugs) in patients with depression, similarly, has been shown to be effective (NNT = 10) but causes considerable adverse events (NNH = 20) [23]. Due to the frequency of adverse side effects, non-adherence to antidepressants is high and associated with decreased remission rates, increased risk of relapse, and increased health care utilization [24]. Hypnotics are frequently prescribed for insomnia. However, they have a poor benefit-to-risk ratio with serious adverse effects including cognitive impairment, injury from falls and automobile accidents (including in younger individuals), cancer, suicide, and hypnotic withdrawal insomnia [25, 26]. In light of this evidence, interest in adjuvant and alternative therapies, especially exercise, has increased in the last decade.

### Description of the intervention

The effects of exercise on depressive symptoms have been summarized in multiple meta-analyses [27–38]. Systematic reviews found moderate-to-large effect sizes for aerobic [28], resistance [36] as well as yoga [35] exercises on depression. Moreover, no significant differences between these interventions and antidepressant medication were found [28, 35]. The effect of other meditative movement exercises such as qi gong and tai chi seems to be positive, albeit less pronounced [37, 38]. Aerobic exercise interventions in depressive patients have also been found to improve cardiorespiratory fitness [27]. This is relevant because depression is known to increase the risk of cardiovascular mortality and morbidity [29, 30].

Current data suggest that exercise might be a suitable therapeutic option to improve sleep quality. Aerobic exercise has been shown to have positive acute (during the night immediately following exercise) and chronic (over several weeks) effects on sleep in healthy individuals with small-to-moderate effect sizes [39, 40]. These findings have been replicated in populations with sleep complaints [39, 41–43] and chronic disorders [44–49] and confirmed by a meta-analysis of previous meta-analyses [50]. A recent meta-analysis also found moderate-to-large effect sizes for mainly chronic resistance training on sleep quality [51]. Lastly, numerous meta-analyses show a positive effect of meditative movement on sleep quality in a variety of patient [49, 52] and elderly [53] populations. However, to the knowledge of the authors, no systematic review concerning the effect of exercise on sleep in patients with depression has been performed.

### Potential mechanisms of action

Although the etiology of insomnia (with or without comorbidities) is not yet fully understood, hyperarousal is widely considered a causal and maintaining factor [54–56].

Multiple mechanisms of action, including ones which involve hyperarousal, have been proposed to explain the effect exercise has on sleep (confer the reviews of Buman and King (2010) [57] and Uchida et al. 2012 [58] for aerobic exercise). Insomniacs have been shown to have impaired thermoregulation [59]. Chronic exercise, on the other hand, improves thermoregulation [60, 61]. Increased skin temperature, which occurs during and immediately after acute aerobic [62], resistance [63], and meditative movement [61, 64] exercise, seems to modulate neural circuits in a way which might be conducive to sleep [65]. Exercise causes changes in the levels of pro-inflammatory cytokines [66], growth hormone [67, 68], and brain-derived neurotrophic factor [69–71] which seem to play a role in the regulation of sleep [72–74]. Aerobic [28], resistance [51], and meditative movement [75] exercise have positive effects on anxiety as well as depression and might thereby reduce psychophysiological arousal. Although it is not fully understood why humans sleep, one hypothesis states that humans sleep to optimize restorative processes [76]. Aerobic and resistance exercise increase energy expenditure and require muscle repair, thus stimulating such restorative processes. Aerobic exercise has also been shown to consistently produce phase shifts (i.e., changes in circadian rhythm within the 24 h cycle) in individuals of different ages and fitness levels. This effect has been found in individuals irrespective of age and cardiorespiratory fitness as well as independent from the effect of light. [77]. Therefore, aerobic exercise may act as a so-called ‘zeitgeber’ positively affecting entrainment (i.e., the synchronization of the endogenous and exogenous rhythms). It should be noted that it is unclear whether the mechanisms of action differ between insomniacs with and without psychiatric comorbidity.

### Why it is important to do this review

The rationale for this review can be summarized in four points. (1) Sleep disturbances are of high prognostic relevance for remission in depression [11]. (2) Current therapies have a dissatisfactory benefit-to-risk-ratio. (3) Exercise has been shown to have positive effects on depression [28, 36, 75] as well as sleep [39, 41, 49, 51, 53]. (4) To the best of our knowledge, no systematic review has been performed to ascertain the effects of aerobic, resistance, and meditative movement exercise on sleep in people with depression.

The main objective of this review is to assess the effects of aerobic, resistance, and meditative movement exercise on sleep quality in patients with depression. A secondary goal is to ascertain the effects of exercise on sleep duration, sleepiness, daytime functioning, use of hypnotics, and adverse events (e.g., injuries, cardiovascular incidences).

## Methods

Before initiation of the project, a search in relevant databases (including PROSPERO) showed no prior or ongoing systematic review of this subject. This systematic review protocol has been reported according to the Preferred Reporting Items for Systematic Review and Meta-analysis Protocols (PRISMA-P) guidelines [78] (see Additional file 1). Accordingly, the protocol for this study was published in the International Prospective Register of Systematic Reviews database (PROSPERO) [79] on 13th February 2019 (PROSPERO CRD42019115705). Should any amendments to this protocol be necessary, they will be documented on the PROSPERO platform. The systematic review and network meta-analysis itself will be presented according to the PRISMA Extension Statement for Reporting of Systematic Reviews Incorporating Network Meta-analyses of Health Care Interventions [80].

### Eligibility criteria

#### Population

Only studies on adult humans (>= 18 years old) of either sex with either a medical diagnosis of unipolar depression or presence of significant depressive symptoms as determined by a validated instrument (e.g., Beck Depression Inventory [81], Research Diagnostic Criteria [82], International Classification of Disease [7], or Diagnostic and Statistical Manual of Mental disorders [6]) will be included. Studies will be excluded if subjects had another substantial somatic disorder which might cause the depressive symptoms (i.e., primary symptoms are not depression) or if subjects were working night-shifts.

#### Intervention

Included trials must allocate subjects to at least one of the following: aerobic, resistance, or meditative movement exercise intervention. Aerobic exercise is defined as “any exercise that primarily uses the aerobic energy-producing systems, can improve the capacity and efficiency of these systems, and is effective for improving cardiorespiratory endurance” [83]. Resistance exercise is defined as “is exercise that causes muscles to work or hold against an applied force or weight” [84]. Meditative movement exercise is defined as a combination of some form of movement or body positioning, breathing, and relaxation [85]. The intervention can be acute (a single bout of exercise) or chronic (repeated exposure). We have not placed restrictions on the duration of the intervention period in order to include the maximum number of trials in this review. Potential statistical heterogeneity or inconsistency due to this factor will be explored (see below). No restrictions are placed on the setting (e.g., laboratory, outdoors), the social context (e.g., individual, group), or the level of supervision (e.g., not guided, under the supervision of an exercise professional). Exercise can be part of a multicomponent intervention. Multicomponent interventions in which exercise was not a dominant part (i.e., exercise was one of four or more intervention modules) will be excluded.

#### Comparison

Trials have to allocate participants to aerobic, resistance, or meditative movement exercise vs. a comparison group. There are no restrictions on the comparison group (e.g., pharmacotherapy, psychotherapy, other exercise intervention).

#### Outcomes

Included trials must measure the effect of aerobic, resistance, or meditative movement exercise on sleep quality. This can be operationalized using self-reports or observer ratings.

#### Study type

In order to be eligible, trials must have employed randomized allocation.

#### Publication status

Studies are included regardless of whether or not they are published in a peer-reviewed journal. The use of unpublished trials in reviews is a controversial topic. Reviews have found that exclusion of gray literature may lead to an overestimation of effect size [86, 87]. On the other hand, van Driel et al. (2009) have shown that unpublished trials have poor or unclear methodological quality [88]. Therefore, methodological quality is considered when deciding whether the network meta-analysis is valid and if the number of studies allows it, subgroup analyses will include methodological quality.

#### Language

Articles written in English or German will be included. Articles in any other language will be included if a translation is made available. Any article which might be relevant, but could not be included due to the aforementioned language constraints will be listed in an appendix.

### Information sources

Multiple sources will be used in this systematic review. A systematic computerized search will be performed in the following online databases: PubMed (on PubMed.gov), EMBASE (on Ovid), Cochrane Library (on cochranelibrary-wiley.com), PsycINFO (on Ovid), SPORTDiscus (on EBSCOhost), and CINHAL (on EBSCOhost). OpenGrey (on opengrey.eu) and ProQuest Dissertations and Theses A&I (on proquest.com) will be searched to include gray literature. Bibliographies of all included studies as well as any other relevant reviews identified via the search will be screened. Clinicaltrials.gov and WHO International Clinical Trials Registry will be searched in order to identify ongoing as well as unpublished studies. Due to lack of controlled vocabulary and restricted length of search strings on these websites, a modified query will be used. Authors of included studies will be contacted via e-mail in order to inquire whether they know of any other relevant publications. All databases will be searched from their inception to the search date.

### Search strategy

The search strategy will be constructed using the PICOS (patient, intervention, comparison, outcome, study design) framework. The search string will be comprised of controlled vocabulary whenever possible and free text. These terms (including appropriate truncation) will be selected in an iterative scoping search using the PICOS approach as well as backward and forward chaining. The study design component will be identified using the “Cochrane highly sensitive search strategies for identifying randomized trials” [89] and translated according to the database. Terms within each group will be combined with a Boolean “OR” and groups will be combined using a Boolean “AND” command. The PubMed search strategy (see Additional file 2) was adapted according to the controlled vocabulary in each database (see Additional file 2). The search strategy has been reviewed by an information scientist from the Basel Medical University Library using the Peer Review of Electronic Search Strategies (PRESS) guideline [90]. Test searches have been performed in order to ensure the validity of the search string.

### Study records

#### Data management

All records identified in the databases will be collected in the reference management software EndNote® X8 (Thomson Reuters, New York, NY). However, deduplication will be performed using the Systematic Review Assistant-Deduplication Module. This software has been shown to have superior sensitivity and specificity in the deduplication process when compared with EndNote [91].

#### Selection process

Upon deduplication, records will be screened in two stages. Firstly, the title and the abstract of all records will be screened against the aforementioned inclusion and exclusion criteria (possible assessments: no (an exclusion criterion is found in title or abstract), maybe or yes (inclusion and exclusion cannot be definitively assessed or study is deemed to fulfill all criteria). Secondly, full texts of all articles which were not excluded in the first stage will be reviewed to determine whether all relevant criteria are met. Both stages will be performed independently by two reviewers (GB and TZS) who will not be blinded to any information (e.g., author, journal, institutions). We do not blind the reviewers, since there is empirical evidence that blinding has little to no effect in meta-analyses [92]. Disagreement will be resolved by consensus. If no consensus can be reached, disagreement will be resolved by adjudication of a designated third reviewer (AST). An online systematic review software, Covidence [93], will be used to judge eligibility, resolve issues, and document the screening processes.

Before the actual screening process begins, both reviewers will screen 50 randomly selected articles in order to assure an adequate inter-rater agreement (Cohen’s kappa > 0.80). Should this goal not be reached, this process will be repeated until the defined level of agreement is reached. Inter-rater agreement will be reported using raw agreement in percent and Cohen’s kappa since both have respective strengths and limitations [94]. Furthermore, the number of disagreements solved by discussion and arbitration by the third reviewer will be stated. A flow diagram according to the PRISMA guidelines [95] will illustrate the number and the reasons for excluded and included citations.

#### Data collection process

A standardized data extraction form will be created in Excel on the basis of the Cochrane Consumers and Communication Review Group’s data extraction template [96] and the DECiMAL guide [97]. This form will be tested against a subset of studies found in the scoping search and adapted accordingly before data extraction. Both reviewers (GB and TZS) will extract data independently. Authors will be contacted should data be missing. (The corresponding author will initially be contacted via e-mail with one additional reminder e-mail, should there be no response within 2 weeks. Subsequently, the other authors will be contacted). Disagreement will be resolved by consensus upon consulting the original paper or if no consensus can be reached, disagreement will be resolved by adjudication of a designated third reviewer (AST). To avoid the inclusion of double publications of one study, authors, treatment comparisons, sample sizes, and outcomes of the included studies will be compared. We will include the publication which has the most information pertinent to the meta-analysis.

### Data items

For the calculation of relative treatment effects group means, corresponding standard deviations and group sizes will be extracted primarily. In case one of these values was missing, other statistical data that can be converted into means and standard deviations will be extracted. Conversions will be calculated according to formulas provided, e.g., [98, 99]. If standard deviations cannot be calculated from the available study information, we will impute them using the standard deviations reported in the other included studies [100]. We will conduct sensitivity analyses excluding studies in which standard deviations had to be imputed. If the *N* was missing in the table of analysis, we will use the *N* of the descriptive statistics. If studies report medians and interquartile ranges, a normal distribution will be assumed, if not indicated otherwise, to convert these values to means and standard deviations [98]. If studies only report adjusted outcome values, data will be extracted, but sensitivity analyses will be calculated without these studies to check for possible bias. We plan to extract the effect size provided by the study authors only if no other information was available for effect size calculation. If it is not possible to impute appropriate measures for the calculation of effect sizes, and no effect sizes are reported we will contact the authors.

Among others, the following information will be extracted from each study:

- Information on the study itself (e.g., title, publication date, authors)
- Methods (e.g., objective, design, number of participants included in the analysis)
- Risk of bias assessment (Cochrane revised risk of bias tool) [101]
- Setting (non-clinical vs. clinical, inpatient vs. outpatient)
- Participants (i.e., mean age, inclusion and exclusion criteria, severity of depression, diagnostic tool)
- Intervention (i.e., frequency, intensity, duration, type of exercise)
- Comparisons (comparator conditions)
- Outcomes (primary and secondary outcomes, adverse events)
- Results (mean and standard deviation of outcomes pre- and post-intervention as well as follow-up)
- Self-report vs. observer rating
- Duration of follow-up

### Outcomes and prioritization

The primary outcome will be standardized mean differences (SMD) of sleep quality at post-exercise-intervention and at the last available follow-up assessment, measured by self-reports (e.g., PSQI [102], ISI [103]) or clinician ratings (sleep-related HAM-D items [104]).

Secondary outcomes will be:

1. SMD of sleep duration at post-exercise intervention and at last available follow-up assessment (measured objectively or subjectively)
2. SMD of daytime functioning at post-exercise intervention and at last available follow-up assessment, measured by self-reports (e.g., Insomnia impact scale [105])
3. SMD of sleepiness at post-intervention and at last available follow-up assessment, measured by self-reports (e.g., Epworth sleepiness scale [106])
4. SMD of hypnotics use at post-intervention and at last available follow-up assessment, measured by self-reports
5. SMD of any adverse events as defined by Good Clinical Practice guidelines [107] (e.g., pain, falls, injuries, dizziness, myocardial infarction)

The rationale for the selection of the primary outcome is that perceived sleep quality, i.e., difficulties initiating or maintaining sleep or early morning awakening is one of the main complaints in insomnia. Reduced sleep duration [108] and daytime impairments are a further important category of complaints, markedly increasing the perceived need for treatment [109]. Adverse events must be considered in order to inform decision-makers on the benefit-to-risk ratio of an exercise intervention.

### Risk of bias in individual studies

The risk of bias will be evaluated independently by two reviewers (GB and TZS) at the study level. Disagreement will be resolved by consensus or if no consensus can be reached, disagreement will be resolved by adjudication of a designated third reviewer (AST). Bias will be assessed using the revised Cochrane risk of bias tool [101]. This tool assesses five domains: (1) randomization process, (2) deviations from intended interventions, (3) missing outcome data, (4) measurement of outcomes, and (5) selection of reported interventions. The three possible judgments are possible: low risk, some concerns, and high risk of bias. A summary table of bias assessment on study level will be included in the publication. These assessments will contribute to the evaluation of overall confidence in the findings of the network meta-analysis using the CINeMA framework [110].

### Data synthesis

Data will be synthesized descriptively. A summary table of included studies will entail information on the authors, population characteristics (diagnostic criteria, baseline severity of sleep quality, depression, age, and numbers), interventions (exposure in each group), outcomes measures used, and results (sleep quality, sleep duration). Network meta-analysis will be performed. Statistical (number of studies and heterogeneity of results), clinical (heterogeneous populations), and methodological (low quality of trials or follow-up duration) aspects will be considered to decide whether network meta-analysis is valid. If network meta-analysis results must be deemed methodologically inaccurate, a pairwise meta-analysis will be considered. Should a pairwise meta-analysis also not be possible, studies will be summarized narratively.

The package netmeta [111] for the open-source software environment R [112] will be used to calculate network meta-analyses within a frequentist framework.

A network will be created including all available jointly randomizable treatments. We assume that any patient that meets all inclusion criteria is likely, in principle, to be randomized to any of the interventions in the synthesis comparator set.

We will address the assumption of transitivity which underlies network meta-analysis [113], by (1) assessing whether the included interventions are similar across studies using a different design, and (2) checking whether the distribution of potential moderators is balanced across comparisons [114]. A priori we have defined depression severity, comorbidities, age, and gender as potential effect modifiers and will evaluate the comparability of the respective characteristics across comparisons qualitatively.

We expect considerable diversity of outcome measures and will, therefore, calculate standardized mean differences (SMD) using Hedge’s *g* with 95% confidence intervals [115]. SMD is the mean difference between groups divided by the pooled standard deviation. The effect size measure allows comparison of effect sizes across similar measurements of a single outcome. The conventional and somewhat arbitrary classification of SMD proposed by Cohen (1988) [116] has been expanded to include very small (.01), small (0.2), medium (0.5), large (0.8), very large (1.2), and huge (2.0) effect sizes [117]. Random-effects pairwise SMDs across studies will be calculated based on the available comparisons between treatment and comparator treatments [118]. Inverse variance weighting is used for pooling. In addition, indirect evidence will be estimated using the entire network of evidence. Random-effects netmeta accounts for dependencies between comparisons in case of multi-arm trials [119]. The command pairwise will be used in case of multi-arm trials, in order to transform the dataset to the comparison level, which is needed for conducting the network meta-analysis.

The primary outcome will be SMD of sleep quality assessed via self- or observer-reported measures. If more than one primary outcome is reported, the most frequently used scale will be included in the analysis to reduce between-study heterogeneity. If possible, we will assess the association between instruments and changes in sleep quality. Two individual analyses will be run for the outcome data at the end of treatment, and the last available follow-up. Separate network meta-analyses will be conducted for secondary outcomes if possible. Results from network meta-analysis will be presented as summary SMD for each possible pair of treatments. Whenever possible, measures of uncertainty will be reported in the form of the 95% confidence interval and 95% prediction interval.

To calculate statistical heterogeneity between studies on the pairwise level, the *Q* statistic will be used [89]. Further *τ*^2^ will be analyzed to estimate the variance caused by the distribution of the true study means [120]. *I*^2^ will be evaluated to indicate the amount of observed variance that can be attributed to between-study heterogeneity [121]. *I*^2^ and the corresponding confidence interval can be interpreted as the percentage of overall heterogeneity that is due to variation of the true effects. An *I*^2^ value of 0% to 40% might not be important, 30 to 60% may represent moderate heterogeneity, 50 to 90% may represent substantial heterogeneity, and 75 to 100% considerable heterogeneity [89]. In NMA, we will assume a common estimate for the heterogeneity variance across the different comparisons.

Local and global methods will be used to detect inconsistency [122]. The presence of inconsistency will be evaluated using the following approaches: (1) locally using the netsplit command (i.e., testing the difference between estimates derived from direct evidence and estimates derived from indirect estimates for statistical significance) and (2) globally using the decomp.design command (i.e., using the design-by-treatment interaction model). For this purpose, the total *Q* statistic (i.e., the measure of total heterogeneity/inconsistency in the network) will be decomposed to an inconsistency factor (between designs) and a heterogeneity factor (within designs). We will compare the magnitude of heterogeneity between consistency and inconsistency models to determine how much heterogeneity will be explained by inconsistency. We will do this by testing the residual inconsistency, which remains under the assumption of a full design by treatment interaction model for statistical significance.

In the case of statistical heterogeneity or inconsistency between results from individual studies, we will investigate the potential impact of the following trial-level effect modifiers: (1) year of publication, (2) study precision (i.e., sample size), (3) studies reporting non-adjusted vs. adjusted means, (4) studies with imputed standard deviations vs. studies which reported standard deviations. If the number of studies allows it, theoretically driven subgroup analyses will be done according to population (e.g., severity of depression), duration of intervention, duration of follow-up, outcome characteristics (i.e., self- vs. observer ratings, objective vs. subjective sleep duration), and methodological quality.

### Meta-biases and confidence in cumulative evidence

The confidence in the network meta-analyses will be estimated using the Confidence in Network Meta-Analysis (CINeMA) framework [110]. This includes study limitation, indirectness, inconsistency (heterogeneity, incoherence), imprecision, and publication bias. Publication bias will be assessed according to the GRADE guideline [123] and by comparing eligible trials identified in registries (e.g., clinicaltrials.gov) with published data. Selective reporting bias will be assessed by comparing protocols (if available) and reports of trials.

### Dissemination

The results will be published in a peer-reviewed journal and presented at conferences as well as invited talks.

## Discussion

This systematic review will provide an overview of the current state of evidence concerning the effects of aerobic exercise on sleep in patients with depression. To the best of our knowledge, this will be the first systematic review concerning this topic. The primary outcomes analyzed will provide evidence on the benefits, i.e., duration and perceived quality of sleep, as well as serious harms. Secondary outcomes will provide information on sleep-related constructs such as daytime functioning and sleepiness as well as other adverse outcomes. Furthermore, gaps in the current literature will be identified, and recommendations for future avenues of research will be given. Strengths of this systematic review include the search in multiple databases according to the interdisciplinary nature of the subject, the systematic approach including screening, data extraction, and quality assessment by two independent reviewers, as well as transparency in reporting according to guidelines. The main limitation is the language restriction to German and English which might lead to language bias. Considering the importance of sleep disturbances in depression, we hope that this systematic review can accelerate the consolidation of evidence, such that decision-makers (patients, health-care professionals, and policy-makers) are provided with high-quality evidence to facilitate decisions on whether and how to implement aerobic, resistance, or meditative movement exercises as a treatment module for patients with depression.

### Current stage of systematic review

PROSPERO stage 1, preliminary searches completed.

## Additional files

Additional file 1: Completed PRISMA-P Checklist. (PDF 345 kb)

Additional file 2: Search strategy for PubMed, EMBASE, PsycINFO, Cochrane Library, SportDiscus, CINAHL, OpenGrey, ProQuest Dissertations and Theses, Clinicaltrials.gov, and International Clinical Trials Registry Platform. (PDF 277 kb)

## Abbreviations

CINeMA: Confidence in Network Meta-Analysis
PICOS: Patient, intervention, comparison, outcome, study design
PRESS: Peer Review of Electronic Search Strategies guideline
PRISMA-P: Preferred Reporting Items for Systematic Reviews and Meta-Analyses for systematic review protocols
PROSPERO: International prospective register of systematic reviews
SMD: Standardized mean difference
