# Supplementary material for: The effects of aerobic, resistance, and meditative movement exercise on sleep in individuals with depression: protocol for a systematic review and network meta-analysis
Source: Syst Rev. 2019 Apr 26;8:105. doi: 10.1186/s13643-019-1018-4 (PMC6486698; doi:10.1186/s13643-019-1018-4)
Supplement: Supplementary file 2 — Search strategy for PubMed, EMBASE, PsycINFO, Cochrane Library, SportDiscus, CINAHL, OpenGrey, ProQuest Dissertations and Theses, Clinicaltrials.gov, and International Clinical Trials Registry Platform. (PDF 277 kb) [file 13643_2019_1018_MOESM2_ESM.pdf]

Additional file 2: Search strategy

PubMed

Patient	"Mood Disorders"[Mesh] OR mood disorder*[tw] OR depression*[tw] OR depressive*[tw] OR depressed*[tw] OR affective disorder*[tw] OR dysthym*[tw]
Intervention	"Exercise"[Mesh] OR exercise*[tw] OR exercising[tw] OR "Exercise Therapy"[Mesh] OR "Physical Fitness"[Mesh] OR "Qigong"[Mesh] OR "Tai Ji"[Mesh] OR "Yoga"[Mesh] OR "Resistance Training"[Mesh] OR aerobic activit*[tw] OR physical activit*[tw] OR sport*[tw] OR walk*[tw] OR run*[tw] OR jog*[tw] OR swim*[tw] OR cycling[tw] OR bicycl*[tw] OR physical training*[tw] OR danc*[tw] OR Tai Chi[tw] OR taichi [tw] OR Taiji [tw] OR Tai Ji[tw] OR Tai-Ji[tw] OR Tai Ji[tw] OR Taijiquan[tw] OR T'ai Chi[tw] OR Yoga [tw] OR Qigong [tw] OR Qi-gong [tw] OR qi gong [tw] OR chi gong [tw] OR ch'i kung [tw] OR Baduanjin [tw] OR mind-body exercise [tw] OR meditative movement[tw] OR Resistance[tw] OR pilates[tw] OR strength[tw]
Outcome	"Sleep"[Mesh] OR Sleep*[tw] OR "Sleep Medicine Specialty"[Mesh] OR "Sleep Disorders, Circadian Rhythm"[Mesh] OR "Sleep Initiation and Maintenance Disorders"[Mesh] OR insomnia*[tw]
Study Design	randomized controlled trial[pt] OR controlled clinical trial[pt] OR randomized[tiab] OR placebo[tiab] OR drug therapy[sh] OR randomly[tiab] OR trial[tiab] OR groups[tiab] NOT (animals [mh] NOT humans [mh])

EMBASE (on Ovid)

Patient	exp mood disorder/ OR mood disorder$.tw. OR depression$.tw. OR depressive$.tw. OR depressed.tw. OR affective disorder$.tw. OR dysthym$.tw.
Intervention	exp exercise/ OR (exercise$ OR exercising).tw. OR exp kinesiotherapy/ OR exp fitness/ OR aerobic activit$.tw. OR exp physical activity/ OR exp sport/ OR exp Yoga/ OR muscle strength/ OR aerobic activit$.tw. OR physical activit$.tw. OR walk$.tw. OR run$.tw. OR jog$.tw. OR swim$.tw. OR (cycling OR bicycl*).tw. OR physical training$.tw. OR danc$.tw. OR Tai Chi.tw. OR taichi.tw. OR Taiji.tw. OR Tai Ji.tw. OR Tai-Ji.tw. OR Taijiquan.tw. OR Yoga.tw. OR Qigong.tw. OR Qi-gong.tw. OR qi gong.tw. OR chi gong.tw. OR Baduanjin.tw. OR mind-body exercise.tw. OR meditative movement.tw. OR pilates.tw. OR strength.tw.
Outcome	exp sleep/ OR sleep$.tw. OR exp sleep medicine/ OR exp circadian rhythm sleep disorder/ OR exp sleep disorder/ OR insomnia$.tw.
Study Design	(crossover-procedure/ or double-blind procedure/ or randomized controlled trial/ or single-blind procedure/ or (random$ or factorial$ or crossover$ or cross over$ or placebo$ or (doubl$ adj blind$) or (singl$ adj blind$) or assign$ or allocat$ or volunteer$).tw.))

Cochrane Library (on cochranelibrary-wiley.com)

Patient	MeSH descriptor: [Mood Disorders] explode all trees OR "mood disorder":ti,ab,kw OR depression*:ti,ab,kw OR depressive*:ti,ab,kw OR depressed:ti,ab,kw OR affective disorder:ti,ab,kw OR dysthym*:ti,ab,kw
Intervention	MeSH descriptor: [Exercise] explode all trees OR (exercise* OR exercising):ti,ab,kw OR MeSH descriptor: [Exercise Therapy] explode all trees OR MeSH descriptor: [Physical Fitness] explode all trees OR MeSH descriptor: [Qigong] explode all trees OR MeSH descriptor: [Tai Ji] explode all trees OR MeSH descriptor: [Yoga] explode all trees OR MeSH descriptor: [Resistance Training] explode all trees OR aerobic activit*:ti,ab,kw OR physical activit*:ti,ab,kw OR sport*:ti,ab,kw OR walk*:ti,ab,kw OR run*:ti,ab,kw OR jog*:ti,ab,kw OR swim*:ti,ab,kw OR (cycling OR bicycl*):ti,ab,kw OR physical training:ti,ab,kw OR danc*:ti,ab,kw OR Tai Chi*:ti,ab,kw OR taichi*:ti,ab,kw OR Taiji*:ti,ab,kw OR Tai Ji*:ti,ab,kw OR Tai-Ji*:ti,ab,kw OR Taijiquan*:ti,ab,kw OR T'ai Chi*:ti,ab,kw OR Yoga*:ti,ab,kw OR Qigong*:ti,ab,kw OR Qi-gong*:ti,ab,kw OR qi gong*:ti,ab,kw OR chi gong*:ti,ab,kw OR ch'i kung*:ti,ab,kw OR Baduanjin*:ti,ab,kw OR mind-body exercise*:ti,ab,kw OR meditative movement*:ti,ab,kw OR Resistance*:ti,ab,kw OR pilates*:ti,ab,kw OR strength*:ti,ab,kw
Outcome	MeSH descriptor: [Sleep] explode all trees OR Sleep*:ti,ab,kw OR MeSH descriptor: [Sleep Medicine Specialty] explode all trees OR MeSH descriptor: [Sleep Disorders, Circadian Rhythm] explode all trees OR MeSH descriptor: [Sleep Initiation and Maintenance Disorders] explode all trees OR insomnia*:ti,ab,kw
Study Design	-

PsycINFO (on Ovid)

Patient	exp MAJOR DEPRESSION/ OR mood disorder*.tw. OR depression$.tw. OR depressive$.tw. OR depressed.tw. OR affective disorder$.tw. OR dysthymia$.tw. OR dysthymic disorder$.tw.
Intervention	exp EXERCISE/ OR (exercise$ OR exercising).tw. OR exp physical fitness/ OR aerobic activit$.tw. OR exp physical activity/ OR exp sports/ OR exp Yoga/ OR exp physical strength/ OR aerobic activit$.tw. OR physical activit$.tw. OR walk$.tw. OR run$.tw. OR jog$.tw. OR swim$.tw. OR (cycling OR bicycl$).tw. OR physical training$.tw. OR exp dance/ OR danc$.tw. OR Tai Chi.tw. OR taichi.tw. OR Taiji.tw. OR Tai-Ji.tw. OR Tai Ji.tw. OR Taijiquan.tw. OR Yoga.tw. OR Qigong.tw. OR Qi-gong.tw. OR qi gong.tw. OR chi gong.tw. OR Baduanjin.tw. OR mind-body exercise.tw. OR meditative movement.tw. OR OR pilates.tw. OR strength.tw.
Outcome	exp SLEEP/ OR sleep$.tw. OR exp sleep treatment/ OR exp sleep disorders/ OR insomnia$.tw.
Study Design	treatment effectiveness evaluation/ or exp Treatment Outcomes/ or placebo/ or exp Followup Studies/ or placebo$.tw. or random$.tw. or comparative stud$.tw. or (clinical adj3 trial$).tw. or (research adj3 design).tw. or (evaluat$ adj3 stud$).tw. or (prospectiv$ adj3 stud$).tw. or ((singl$ or doubl$ or trebl$ or tripl$) adj3 (blind$ or mask$)).tw.

SportDiscus (on EBSCOhost)

Patient	DE "AFFECTIVE disorders" OR DE "MENTAL depression" OR TI "mood disorder*" OR AB "mood disorder*" OR TI depression* OR AB depression* OR TI depressive* OR AB depressive* OR TI depressed OR AB depressed OR TI "affective disorder*" OR AB "affective disorder*" OR TI dysthym* OR AB dysthym*
Intervention	DE "EXERCISE" OR DE "EXERCISE" OR TI (exercise* or exercising) OR AB (exercise* or exercising) OR DE "EXERCISE therapy" OR DE "PHYSICAL fitness" OR TI "aerobic activit*" OR AB "aerobic activit*" OR DE "PHYSICAL activity" OR DE "SPORTS" OR TI walk* OR AB walk* OR TI run* OR AB run* OR TI jog* OR AB jog* OR TI swim* OR AB swim* OR TI (bicycl*) OR AB (bicycl*) OR TI "physical training" OR AB "physical training" OR TI (danc*) OR AB (danc*) OR TI (Tai Chi) OR AB (Tai Chi) OR TI (taichi) OR AB (taichi) OR TI (Taiji) OR AB (Taiji) OR TI (Tai Ji) OR AB (Tai Ji) OR TI (Tai-Ji) OR AB (Tai-Ji) OR TI (Taijiquan) OR AB (Taijiquan) OR TI (T'ai Chi) OR AB (T'ai Chi) OR TI (Yoga) OR AB (Yoga) OR TI (Qigong) OR AB (Qigong) OR TI (Qi-gong) OR AB (Qi-gong) OR TI (qi gong) OR AB (qi gong) OR TI (T'ai Chi) OR AB (T'ai Chi) OR TI (chi gong) OR AB (chi gong) OR TI (ch'i kung) OR AB (ch'i kung) OR TI (Baduanjin) OR AB (Baduanjin) OR TI (mind-body exercise) OR AB (mind-body exercise) OR TI (meditative movement) OR AB (meditative movement) OR DE "PILATES method" OR TI (resistance) OR AB (resistance) OR TI (pilates) OR AB (pilates) OR TI (strength) OR AB (strength)
Outcome	DE "SLEEP" OR TI sleep* OR AB sleep* OR DE "SLEEP disorders" OR DE "SLEEP interruptions" OR TI insomnia* OR AB insomnia*
Study Design	PT Clinical trial OR AB random* OR TI random* OR AB placebo or TI placebo OR SU drug therapy OR AB trial OR TI trial OR AB groups or TI groups NOT (SU animals NOT human)

CINHAL (on EBSCOhost)

Patient	(MH "Affective Disorders+") OR TI (mood disorder*) OR AB (mood disorder*) OR TI depression* OR AB depression* OR TI depressive* OR AB depressive* OR TI depressed OR AB depressed OR TI (affective disorder*) OR AB (affective disorder*) OR TI dysthym* OR AB dysthym*
Intervention	(MH "Exercise+") OR TI (exercise* or exercising) OR AB (exercise* or exercising) OR (MH "Therapeutic Exercise") OR (MH "Physical Fitness") OR (MH "Qigong") OR (MM "Tai Chi") OR (MM "Yoga") OR (MH "Muscle Strengthening+") OR TI (aerobic activit*) OR AB (aerobic activit*) OR (MH "Physical Activity") OR (MH "Sports+") OR AB walk* OR TI walk* OR AB run* OR TI run* OR AB jog* OR TI jog* OR AB swim* OR TI swim* OR TI bicycl* OR AB bicycl* OR TI (physical training) OR AB (physical training) OR TI danc* OR AB danc* OR TI Tai Chi OR AB Tai Chi OR TI taichi OR AB taichi OR TI Taiji OR AB Taiji OR TI Tai Ji OR AB Tai Ji OR TI Tai-Ji OR AB Tai-Ji OR TI Taijiquan OR AB Taijiquan OR TI T'ai Chi OR AB T'ai Chi OR TI Yoga OR AB Yoga OR TI Qigong OR AB Qigong OR TI Qi-gong OR AB Qi-gong OR TI qi gong OR AB qi gong OR TI chi gong OR AB chi gong OR TI ch'i kung OR AB ch'i kung OR TI Baduanjin OR AB Baduanjin OR TI mind-body exercise OR AB mind-body exercise OR TI meditative movement OR AB meditative movement OR TI Resistance OR AB Resistance OR TI pilates OR AB pilates OR TI strength OR AB strength
Outcome	(MH "Sleep") OR TI sleep* OR AB sleep* OR (MH "Sleep Disorders, Circadian Rhythm") OR (MH "Sleep Disorders") OR (MH "Insomnia") OR TI (insomnia*) OR AB (insomnia*)
Study Design	PT Clinical trial OR AB random* OR TI random* OR AB placebo or TI placebo OR SU drug therapy OR AB trial OR TI trial OR AB groups or TI groups NOT (SU animals NOT human)

OpenGrey (on opengrey.eu)

Patient	("mood disorder*" OR depression* OR depressive* OR depressed OR "affective disorder*" OR dysthym*)
Intervention	(exercise* OR exercising OR "aerobic activit*" OR "physical activit*" OR sport* OR walk* OR run* OR jog* OR swim* OR cycling OR bicycl* OR "physical training*" OR danc* OR pilates OR “Tai Chi” OR “taichi” OR “Taiji” OR “Tai Ji” OR “Tai-Ji” OR “Taijiquan” OR “T'ai Chi” OR “Yoga” OR “Qigong” OR “Qi-gong” OR “qi gong” OR “chi gong” OR “ch'i kung” OR “Baduanjin” OR “mind-body exercise” OR “meditative movement” OR Resistance OR strength)
Outcome	(Sleep* OR insomnia*))
Study Design	-

ProQuest Dissertations & Theses A&I (on proquest.com)

Patient	(SU(mental depression) OR ti("mood disorder*" OR depression* OR depressive* OR depressed OR "affective disorder*" OR dysthym*) OR ab("mood disorder*" OR depression* OR depressive* OR depressed OR "affective disorder*" OR dysthym*))
Intervention	(SU(exercise OR physical fitness) OR SU(yoga) OR ti(exercise* OR exercising OR "aerobic activit*" OR "physical activit*" OR sport* OR walk* OR run* OR jog* OR swim* OR cycling OR bicycl* OR "physical training*" OR danc* OR pilates OR Tai Chi OR taichi OR Taiji OR Tai Ji OR Tai-Ji OR Taijiquan OR T'ai Chi OR Yoga OR Qigong OR Qi-gong OR qi gong OR chi gong OR Baduanjin OR mind-body exercise OR meditative movement OR Resistance OR strength) OR ab(exercise* OR exercising OR "aerobic activit*" OR "physical activit*" OR sport* OR walk* OR run* OR jog* OR swim* OR cycling OR bicycl* OR "physical training*" OR danc* OR pilates OR Tai Chi OR taichi OR Taiji OR Tai Ji OR Tai-Ji OR Taijiquan OR T'ai Chi OR Yoga OR Qigong OR Qi-gong OR qi gong OR chi gong OR Baduanjin OR mind-body exercise OR meditative movement OR Resistance OR strength))
Outcome	(SU(sleep) OR SU(sleep disorder) OR SU(insomnia) OR ti(Sleep* OR insomnia*) OR ab(sleep* OR insomnia*))
Study Design	-

Clinicaltrials.gov

Patient	Mood disorder OR affective disorder OR depression OR depressive OR depressed OR dysthym*
Intervention	Exercise OR exercising OR physical activity OR aerobic activity OR sport OR walking OR jogging OR swimming OR bicycle OR cycling OR physical training OR dancing OR mind-body OR meditative movement OR qi gong OR tai chi OR yoga OR resistance OR strength OR pilates
Outcome	Sleep OR Circadian Rhythm OR insomnia
Study Design	Interventional Studies

WHO International Clinical Trials Registry

Condition	Mood disorder OR affective disorder OR depression OR depressive OR depressed OR dysthym*
Intervention	Exercise OR exercising OR physical activity OR aerobic activity OR sport OR walking OR running OR jogging OR swimming OR bicycle OR cycling OR dancing OR mind-body OR meditative movement OR qi gong OR tai chi OR yoga OR resistance OR pilates OR strength
Outcome	-
Study Design	-
